# The genome-wide transcription response to telomerase deficiency in the thermotolerant yeast *Hansenula polymorpha* DL-1

**DOI:** 10.1186/s12864-017-3889-x

**Published:** 2017-06-28

**Authors:** Alexey V. Beletsky, Alexander N. Malyavko, Maria V. Sukhanova, Eugenia S. Mardanova, Maria I. Zvereva, Olga A. Petrova, Yulia Yu. Parfenova, Maria P. Rubtsova, Andrey V. Mardanov, Olga I. Lavrik, Olga A. Dontsova, Nikolai V. Ravin

**Affiliations:** 1 Institute of Bioengineering, Research Center of Biotechnology of the Russian Academy of Sciences, Leninsky Ave. 33, bld 2, Moscow, 119071 Russia; 2 Faculty of Chemistry, Moscow State University, Leninskie Gory 1, bld. 3, Moscow, 119991 Russia; 3 Institute of Chemical Biology and Fundamental Medicine, Siberian Branch, Russian Academy of Sciences, Lavrentiev Ave. 8, Novosibirsk, 630090 Russia; 4 Belozersky Institute of Physico-Chemical Biology, Moscow State University, Leninskie Gory 1, bld. 40, Moscow, 119992 Russia; 5 Novosibirsk State University, Novosibirsk, 630090 Russia; 6 Center of Functional Genomics, Skolkovo Institute of Science and Technology, Moscow, 143026 Russia

**Keywords:** Telomerase, Senescence, Yeast, *Hansenula polymorpha*, RNA-seq, DNA repair, Autophagy, Environmental stress response

## Abstract

**Background:**

In the course of replication of eukaryotic chromosomes, the telomere length is maintained due to activity of telomerase, the ribonucleoprotein reverse transcriptase. Abolishing telomerase function causes progressive shortening of telomeres and, ultimately, cell cycle arrest and replicative senescence. To better understand the cellular response to telomerase deficiency, we performed a transcriptomic study for the thermotolerant methylotrophic yeast *Hansenula polymorpha* DL-1 lacking telomerase activity.

**Results:**

Mutant strain of *H. polymorpha* carrying a disrupted telomerase RNA gene was produced, grown to senescence and analyzed by RNA-seq along with wild type strain. Telomere shortening induced a transcriptional response involving genes relevant to telomere structure and maintenance, DNA damage response, information processing, and some metabolic pathways. Genes involved in DNA replication and repair, response to environmental stresses and intracellular traffic were up-regulated in senescent *H. polymorpha* cells, while strong down-regulation was observed for genes involved in transcription and translation, as well as core histones.

**Conclusions:**

Comparison of the telomerase deletion transcription responses by *Saccharomyces cerevisiae* and *H. polymorpha* demonstrates that senescence makes different impact on the main metabolic pathways of these yeast species but induces similar changes in processes related to nucleic acids metabolism and protein synthesis. Up-regulation of a subunit of the TORC1 complex is clearly relevant for both types of yeast.

**Electronic supplementary material:**

The online version of this article (doi:10.1186/s12864-017-3889-x) contains supplementary material, which is available to authorized users.

## Background

Telomeres are the specialized DNA–protein structures at the ends of eukaryotic chromosomes. Telomeres are composed of repetitive DNA elements and proteins that protect eukaryotic chromosomes from end-to-end fusion and uncontrolled degradation, thus contributing to genomic stability. Telomeres are bound by several proteins that prevent recognition of the chromosome ends as double-stranded breaks [1, 2].

Another vital function of telomeres is to solve the problem of incomplete replication of linear chromosomes, which arises due to the inability of DNA polymerases to fully replicate chromosome ends [1]. In eukaryotes, this problem is solved due to telomerase, a special enzyme complex that through the activity of its reverse transcriptase (TERT) uses an RNA template (TER) to add tandem repeats of a simple telomeric DNA sequence to the chromosome ends. Upon synthesis of this extension, the DNA polymerases can replicate the complementary strand, creating a double-stranded DNA molecule [1]. The activity of telomerase in the synthesis of telomeric repeats is typically precisely controlled to maintain the particular length of telomeres. For example, in yeast *Saccharomyces cerevisiae* the length of telomeres is about 350 bp, while human telomeres span several thousand base pairs.

In yeast cells, abolishing the functions of telomerase causes progressive shortening of telomeres with every cell division until they reach a critically short length that promotes cell cycle arrest in the G2/M stage, resulting in replicative senescence of most of the cells in the population [3]. Cell cycle arrest was also observed when telomere damage was caused by the absence of telomere-binding proteins [4, 5]. Particularly, the Cdc13 protein binds single-stranded telomere sequences, thus protecting the ends from degradation, although alternative mechanisms of telomere capping do exist [6–8].

The loss of telomerase activity apparently results in the appearance of “naked” chromosome ends that should be recognized as double-stranded breaks by DNA repair machinery and, finally, in replicative cell senescence [9]. Genome-wide microarray analysis of the transcriptional response of *Saccharomyces cerevisiae* to deletion of the telomerase RNA revealed that telomere shortening is associated with global changes in gene expression that has some similarities to the DNA damage and the environmental stress response pattern, but also possess unique features that suggests that shortened telomeres induce a specific cellular response [10, 11]. For example, telomerase deletion response includes up-regulation of energy production genes, accompanied by a proliferation of mitochondria [10]. In another study [12], the genome-wide response to uncapped telomeres was analyzed in the *S. cerevisiae* cdc13–1 strain, which has a temperature-dependent telomere capping defect. Telomere uncapping was associated with the differential expression of over 600 transcripts; the response was related to, but distinct from, the response to non-telomeric double-strand breaks [12].

We focused our research on a telomerase from the thermotolerant yeast, *Hansenula polymorpha* DL-1, which is able to grow at temperatures up to 50 °C [13]. The telomeres of *H. polymorpha* were reported to consist of the highly regular pattern 5′- GGGTGGCG-3′ repeated 18–23 times [14], and are much shorter than the telomeres in *S. cerevisiae*. Thus, one can expect that the transcriptional response to telomere deficiency in *H. polymorpha* could be more specific due to the lower number of generations required to activate cell senescence, thus, reducing heterogeneity within populations of cells, and higher “resistance” of *H. polymorpha* to environmental stresses that could overlap telomerase-deficiency transcription pattern.

Previous analysis of the subtelomere regions of *H. polymorpha* did not reveal the presence of the repeated elements similar to the Y′ of *S. cerevisiae*. However, short ARS-containing sequences were found at three chromosomal ends, suggesting the presence of elements with the properties of X-sequences of *S. cerevisiae* [15]. Subtelomere regions of *H. polymorpha* are enriched in several groups of metabolic genes, various permeases, and transporters responsible for metal, amino acid, and carbohydrate uptake, redox processes and NADPH regeneration, with the most abundant being the group of MFS membrane transporters [15].

In this paper, we examined genome-wide changes in mRNA transcript levels in *H. polymorpha* DL-1 by transcriptome sequencing after deleting HpTER, the telomerase RNA component. Telomere shortening evoked transcriptional responses involving genes relevant to telomere structure and maintenance, DNA damage response, information processing, and some metabolic pathways.

## Results

### Genome-wide response to deletion of telomerase RNA

**Fig. 1:**
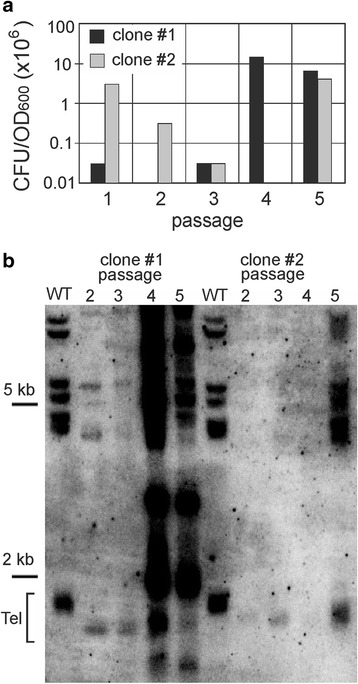
Shortening of telomeres and loss of viability during passaging of *H.polymorpha* D_TER strain. **a** Cell viability test for clones #1 and #2. Viability is expressed as colony forming units (CFU) per unit of OD_600_. **b** Southern blot analysis of telomere-proximal restriction fragments in the wild-type and D_TER strains (clones #1 and #2). The position of DNA marker is shown on the left. Tel – positions of shortest telomere-proximal fragments. Note that clone #1 (passages 4 and 5) and of clone #2 (passage 5) represents telomerase-independent “survivals”

To analyze the genome-wide transcription response to the loss of telomerase activity, a telomerase RNA null mutant (D_TER) was produced after transformation of the wild-type strain with the disruption construct for HpTER gene knockout [16]. Two single colonies of the D_TER strain obtained after transformation (clones #1 and #2) were inoculated into the liquid medium and passaged six times. During passaging, cell viability and telomere length were monitored. Cell viability was lost and telomeres shortened at the earliest time-points (Fig. 1). For clone #2, we estimated the cell cycle distribution of the population and found that after the second passage the majority of cells were in G2/M phase (Additional file 1: Figure S1), consistent with the loss of protective function of telomeres. Such early senescence onset has been previously observed [16], and most likely stems from shorter telomeres in *H. polymorpha* DL-1 compared to *S. cerevisiae* [14]. Thus, it was not possible to perform time-course transcriptome analysis. Cells collected during passages two and three of the clone #1 and passages three and four of the clone #2 were used as biological replicates and were analyzed by RNA-seq, together with two replicates of the wild-type strain.

The expression of 990 of a total 5326 annotated chromosomal genes was changed by two-fold or more relative to the wild-type strain. Of these, 624 (11.7%) were induced, and 366 (6.9%) were repressed (Table 1). These values are higher than observed in the telomerase-deficient *S. cerevisiae* strain in microarray experiments (650 from 6200 analyzed ORFs in [10]) indicating either a difference in telomerase-deficiency responses in these yeast species, a higher sensitivity of the RNAseq approach, or both. Very limited differences in gene expression values were observed between two consecutive passages for each of the two D_TER clones, which is consistent with the early senescence.

**Table 1:** Numbers of up- and down-regulated genes in *H. polymorpha* strains deficient for telomerase complex

Expression level		D_TER
Up-regulated	X > 5	76
	5 > X > 2	548
Unchanged	2 > X > 0.5	4322
Down-regulated	0.5 > X > 0.2	361
	0.2 > X	5

X, relative expression level defined as normalized number of reads mapped to particular gene in mutant strain divided by the same in the control strain. Expression of a total of 5312 genes was detected in the control strain

The genome-wide landscape of the *H. polymorpha* transcriptome in wild-type and D_TER strains revealed that some up-regulated and down-regulated genes tend to form clusters separated by extended regions exhibiting a low variation of expression (Additional file 1: Figure S2).

**Fig. 2:**
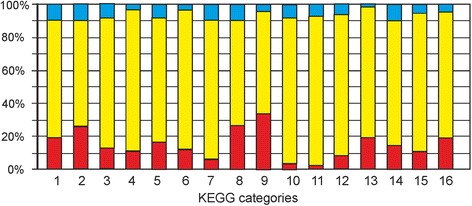
Up-regulation and down-regulation of *H. polymorpha* genes distributed to various KEGG categories in *H. polymorpha* D_TER strain. Metabolism: 1 - Carbohydrate metabolism, 2 - Energy metabolism, 3 – Lipid metabolism, 4 - Nucleotide metabolism, 5 -Amino acid metabolism, 6 - Glycan biosynthesis and metabolism, 7 - Metabolism of cofactors and vitamins, 8 - Biosynthesis of other secondary metabolites, 9 - Xenobiotic biodegradation and metabolism. Genetic Information Processing: 10 - Transcription, 11 - Translation, 12 - Folding, sorting and degradation, 13 - Replication and repair. Environmental Information Processing: 14 - Signal transduction. Cellular Processes: 15 - Transport and catabolism, 16 - Cell growth and death. Fractions of up-regulated genes are shown in *red*, down-regulated in *blue*, and genes without significant changes in expression in *yellow*

The set of differentially expressed genes includes a wide range of functional categories including DNA replication and repair, stress response, transcription and translation, cell wall maintenance, energy production and carbohydrate metabolism. To obtain an integrated view of the pattern of gene expression in wild-type and D_TER strains, we analyzed the expression levels of genes functionally subdivided into KEGG groups and categories. In this analysis, each gene may be classified into one or more groups, depending on its function. The data shown in Fig. 2 indicate the proportions of genes that are upregulated, downregulated, or have similar expression levels in D_TER strain relative to wild-type. As expected, genes assigned to “Replication and Repair” and “Cell Growth and Death” categories were higher expressed in the D_TER strain than wild-type. For example, 19% of genes involved in replication and repair were induced in the D_TER strain, whereas only 1% of these were repressed (Additional file 2: Table S1). The opposite trend was unexpectedly observed for genes involved in transcription (3% induced vs. 9% repressed) and translation (2% induced vs. 7% repressed [in D_TER compared wild-type]). Particularly, among 94 genes assigned to the KEGG category “Ribosome” (03010), 16 genes were repressed more than two-fold, and none were induced in the D_TER strain compared to wild-type. Down-regulation was observed for both cytoplasmic and mitochondrial ribosomal protein genes indicating a global decrease of protein synthesis in senescent telomerase-deficient cells. Such down-regulation of ribosomal protein genes was not observed for *H. polymorpha* grown under heat stress [17] or with methanol [15].

### Known genes involved in telomere maintenance

Among known or suggested components of the telomerase complex, only the HPODL_02050 and HPODL_01499 genes, encoding the Ku70/Ku80 complex that binds to DNA double-strand break ends and directly to telomeric DNA ends, were moderately induced in the D_TER strain (1.5–1.7 fold), while expression of the HPODL_01459 (*EST1*), HPODL_01277 (*EST2*), HPODL_02192 (*EST3*) genes, encoding protein components of the telomerase were not significantly changed (Additional file 2: Table S2). Expression of two other genes previously implicated in telomere function, *CDC13* (HPODL_00415), coding for putative essential telomere binding protein, responsible for telomere end protection [4], and *TBF1* (HPODL_03795), which encodes a DNA-binding protein [18], were not changed. The *STN1* gene (HPODL_03833), encoding Cdc13-interacting telomere end-binding protein was also up-regulated 1.6-fold.

In *S. cerevisiae*, seven Sm- proteins were marked as additional components of the telomerase complex [19] since they are required for the stability of telomerase RNA [20]. Expression of most of the genes encoding the members of the Sm and Lsm family proteins, including *SMB1* (HPODL_00463), *SMD1* (HPODL_01396), *SMD2* (HPODL_00571), *SME1* (HPODL_01477), *SMX2* (HPODL_02984), *SMX3* (HPODL_03956), *LSM1* (HPODL_03359), *LSM3* (HPODL_05140), *LSM4* (HPODL_00774), *LSM5* (HPODL_03344), and *LSM7* (HPODL_04033) was down-regulated.

Different expression patterns were observed for the *RAP1* gene, a central negative regulator of telomere length in *S. cerevisiae* that regulates access of the telomerase to the telomere in a length-dependent manner [19]. BLAST search revealed two candidate homologs of Rap1 that suggests a possible duplication of *RAP1* in *H. polymorpha*, − an expression of one gene (*RAP1A*, HPODL_03159) was not changed, while transcription of the second gene (*RAP1B*, HPODL_04303) was downregulated about two-fold. In *S. cerevisiae*, two sets of proteins interact with Rap1: Sir3 and Sir4 are responsible for the formation of heterochromatin in subtelomeric regions [21], and Rif1 and Rif2 are involved in the regulation of telomere length [22, 23]. *SIR3, SIR4,* and *RIF2* were not identified in the *H. polymorpha* genome, whereas *RIF1* (HPODL_4218) was up-regulated.

### Telomere-proximal genes

Analysis of expression patterns of genes located close to the telomeres of six out of seven *H. polymorpha* DL-1 chromosomes (chromosome 4 consists of two contigs with unknown relative orientation) clearly demonstrated elevated expression of subtelomeric genes in the D_TER strain: expression of about 36% of these genes located within 20 kb of any chromosome end was upregulated at least two-fold relative to wild-type, while only 7% of genes were repressed. This trend became even more pronounced in the 5-kb telomere-proximal window, where 68% of genes were up-regulated at least two-fold, and only 3% of genes were downregulated in the D_TER strain. Induction of subtelomeric genes could result from the loss of silencing of telomere-proximal regions upon dissociation of the telomerase complex and uncapping of telomeres. Similar effects were observed for subtelomeric genes in senescent cells of *S. cerevisiae* [10].

### Autophagy

Telomerase deficiency has an influence on autophagy, which is usually activated by the decrease of amino acid concentration or nitrogen sources in the medium. Deletions in ATG (autophagic-related) genes decrease the period that yeast cells remain viable in a non-dividing state after the arrest of growth [24]. We observed modest upregulation (1.5-fold or more) of several genes involved in autophagy, including autophagy-related protein 21 (HPODL_01040) and putative lipase ATG15 (HPODL_03503) (Additional file 2: Table S3).

There are the three nutrient-sensing pathways regulating autophagy in yeast, − PKA, Sch9, and TORC1 [25]. The main autophagy inhibitor is the TORC1 complex that has kinase activity and inhibits the function of Atg13 kinase by phosphorylation. One gene of this complex in *H. polymorpha* (HPODL_00261) was upregulated 1.90-fold in the D_TER strain. The gene encoding Atg13, the target of TORC1 kinase, was also up-regulated (1.94-fold). Similarly, we observed moderate changes in the expression of genes encoding PKA (HPODL_04656) and Sch9 (HPODL_03332). The PKA–Sch9-mediated autophagy depends on the Rim15 kinase [25], and we detected a 1.75-fold increase of transcription of this gene (*RIM15*, HPODL_04619).

### Cell architecture and intracellular traffic

Telomerase deficiency promotes telomere shortening and leads to cell cycle arrest and replicative senescence of the most cell population. Consequently, cells do not need more core histones to assemble the replicated genome into chromatin. Indeed, we observed down-regulation of expression of genes encoding the major core histones (H3, H4, H2A, and H2B), and centromeric histone H3 in D_TER strain (Additional file 2: Table S4). Expression of the major histone gene-specific transcription activator, *SPT10*, was also down-regulated.

We also observed a two-fold up-regulation of genes encoding tubulin (HPODL_4315 and HPODL_2853, coding of subunits alpha and beta, respectively) and an approximately three-fold increase in expression of dynein (HPODL_3488) in D_TER strain. The tubulin levels per cell increased in *S. cerevisiae* cells at senescence [26], which can be accounted for by the increased volume of senescent cells [10].

### DNA damage–related genes

Deficiency in telomerase apparently results in the appearance of unprotected double-stranded termini of chromosomes that should be recognized and processed by cellular DNA damage signaling or/and DNA repair systems [9]. Consistently, some genes assigned to the KEGG category “Replication and Repair” were up-regulated in the D_TER strain. The loss of integrity of telomeres can activate the DNA damage response (DDR) and elicit DNA repair [27, 28]. There are no data describing changes in the expression of DDR signaling, DNA replication/repair genes upon inactivation of telomerase in *H. polymorpha.* Here, we attempted to determine the possible influence of the inactivation of telomerase complex on the transcription of genes assigned to KEGG categories “Cell Growth and Death”, namely, DNA damage signaling checkpoint proteins, and “Replication and Repair”. The transcription pattern of DNA damage response (DDR) genes, double strand break (DSB) repair via homologous recombination (HR) or non-homologous end joining (NHEJ), base excision repair (BER), nucleotide excision repair (NER), mismatch repair (MMR) and post-replication repair (PRR) were studied in the D_TER strain (Additional file 2: Table S5). Although hundreds of genes are directly or indirectly involved in DDR and DNA repair pathways [29–33], we selected 11 DDR signaling genes associated directly with DNA damage checkpoint response, and the genes coding for major factors related to HR, NHEJ, BER, NER, MMR and PRR pathways of DNA repair in *S. cerevisiae* (Additional file 2: Table S5) [34–36]. Changes in expression of 195 DNA replication/repair and 11 DNA damage signaling checkpoint genes were analyzed via mRNA expression level in wild type and D_TER mutant cells.

Deletion of the gene encoding telomerase RNA had a significant effect on expression of DDR genes. The first step in DDR is the recognition of damaged DNA by sensory proteins including Rad24-RFC_2–5_, Ddc1-Rad17-Mec3, and Mre11-Rad50 complexes, leading to activation of Mec1 and Tel1 kinases that affect cell cycle progression involving protein kinases Rad53, Dun1, and Chk1 [29]. *DDC1*, *RFC*-related, *RAD53*, *CHK1*, and *MRE11* genes changed insignificantly in all mutant strains, but the *RAD17*, *RAD24*, and *RAD50* genes showed an increase of the expression level in the mutant strain (Additional file 2: Table S5). Additionally, other checkpoint genes such as *TEL1* and *DUN1* showed higher expression levels in the D_TER strain than wild-type. It is likely that the elevated expression of proteins involved in DSB recognition, namely Tel1, at the transcriptional level, can be associated with the initial step of DSB-like sensing of telomeres by DDR system in D_TER strain of *H. polymorpha*. Telomere replication may generate a short 3′ overhang and a blunt end in DNA strands synthesized by the lagging and the leading strand synthesis machinery, respectively [37–39]. Similarly to *S. cerevisiae,* the MRX complex recruits Tel1 at blunt, or minimally processed DNA ends, Tel1 can subsequently activate Rad53, Mrc1, and Chk1 to establish transient cell cycle arrest, the so-called pre-senescence state [28, 40–43].

Some DSB repair proteins, including Ku70/80, Mre11/Rad50/Xrs2, DNA helicase Sgs1, and endonuclease Sae2, are implicated in processing telomeres in normal cells [35, 44]. Interestingly, the changes in expression of DSB repair-related genes were clearly observed only for HR process, while the transcription of genes involved in NHEJ, such as *KU70/KU80* and *LIG4*, was less than 2-fold upregulated in the D_TER mutant (Additional file 2: Table S5). In the case of genes related to HR process, the mRNA levels of the *EXO1, RAD51, RAD52, RAD54, SLX1, MUS81, SGS1,* and *TOP3* genes were elevated in the mutant. The nuclease and helicase activities encoded by the HR genes can be involved in the processing of telomeres, similar to the initial response to DSBs repair in *S. cerevisiae* [35]. At the same time, the HR factors, such as Exo1, Rad51, Rad52, and Sgs1 are critical for the survival of telomerase-negative cells [19, 45, 46].

Interestingly, most of the genes encoding NER proteins seem to be unaffected in the D_TER strain. Of 20 tested genes, only three (*RAD4, RAD16,* and *HPODL_02181*) have shown more than a two-fold increase in expression level, and one gene was down-regulated more than two-fold in the D_TER strain compared to wild type (Additional file 2: Table S5).

**Fig. 3:**
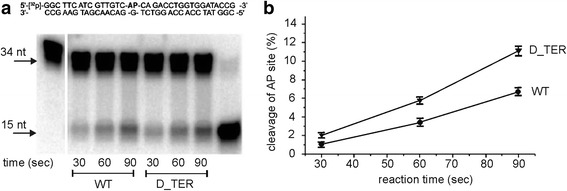
Analysis of apurinic/apyrimidinic (AP) endonuclease activity in wild-type and D_TER strains. **a** PAGE analysis of 5′-[^32^P]-labelled AP site-containing DNA duplex upon incubation with protein extracts from wild-type and D_TER strains. The reaction mixtures were incubated at 37 °C for 30, 60 and 90 s. Processing of AP site resulted in the appearance of ~15 nt band. The nucleotide sequence of the target DNA duplex is shown in the *upper part*. **b** Quantitative analysis of the results is shown in panel A. The cleavage efficiency was defined as the percentage (%) of 15-nt product formation, which was calculated as the ratio of 15-nt products to the sum of all products identified on the gel. The values represent the average of three independent measurements

In the case of the BER system, the expression of apurinic/apyrimidinic endonuclease genes (*APN1* and *APN2*) were found to be upregulated in the mutant strain, while 8-OxoGuanine Glycosylase (*OGG1*), uracil-DNA glycosylase (*UNG*), and DNA-3-methyladenine glycosylase II (*MAG1*) were unchanged. Both Apn1 and Apn2 are considered as major apurinic/apyrimidinic endonuclease in yeast cells [36]. After detecting significant changes in Apn1(2) at the mRNA levels, we examined the apurinic/apyrimidinic (AP) site incision activity in D_TER compared to the wild-type strain. We used DNA duplexes containing a single AP site (DNA-AP) and analyzed the processing of these DNA lesions by the cell extracts. We found that the level of AP site incision activity is slightly increased in the cell extracts produced from telomerase-deficient cells (Fig. 3). Recently, the key role of Ape1, mammalian functional homologue of yeast Apn1, in protection of telomeres was established in human cells [47]. Our results suggest that adaptation of *H. polymorpha* cells to the loss of telomerase may include up-regulation of BER-related genes *APN1* and *APN2*.

We also monitored the changes of transcription of DNA replication genes in telomerase mutant cells. Out of 28 genes detected, only two appeared to be upregulated in the D_TER strain more than two-fold, and none were down-regulated (Additional file 2: Table S5). It is remarkable that transcription of the gene encoding the large subunit of the replication factor A (RPA1) was significantly induced. Single-strand DNA-binding protein, RPA, contributes to DNA replication and this protein is involved in checkpoint signaling, as well as in NER systems and HR in assembly with the Rad51 and Rad52 proteins [48].

It is known that upon the cultivation of *S. cerevisiae* strains without telomerase activity, a subpopulation of “survivors” become selected [3]. The telomerase-negative cells can overcome the replicative senescence via telomere elongation through recombinational telomere elongation (RTE) [3, 49, 50]. *S. cerevisiae* has two recombination-dependent pathways to complete telomere replication in telomerase-lacking cells, the Rad52 and Rad51-dependent (type I) or Rad52 and Rad50-dependent, (Rad51-independent, type II) RTE pathways [19, 51]. The type I pathway depends on the “canonical” HR proteins and requires Rad51, Rad54, Rad55, and Rad57, the second pathway is close to Rad51-independent break-induced replication (BIR) and requires Rad52, helicase Sgs1, Top3, the Mre11-Rad50 complex, and Rad59 [52, 53]. However, the distinction between type I and type II survivors is not fully understood [54]. At the level of transcription, HR genes closely related to both types of RTE pathway, including *RAD51* and *RAD52*, showed upregulation in the mutant strains (Additional file 2: Table S5). The up-regulation of BIR-related genes, such as *SGS1, POL32, MCM2–7,* and *TOP3*, appears to correlate with activation of the type II pathway in “survivor” D_TER cells [53]. We also observed a moderate upregulation of the *RAD50* gene that plays a major role in the generation of type II survivors [20]. The HR pathway is likely to be one of the main mechanisms involved in elongation of telomeres in the telomerase-deficient cells [55–57]. Therefore, the changes observed in this pathway were not unexpected, although the alteration of mRNA levels of some genes related to BER, NER, MMR, and PRR in the mutant strain was not anticipated and warranted further study of the adaptation of *H. polymorpha* cells to telomerase inactivation.

### Environmental stress response

Similar to *S. cerevisiae* [10, 11], deficiency of telomerase in *H. polymorpha* DL-1 results in the induction of a number of environmental stress-response genes (Additional file 2: Table S6). Notably, expression of most of the heat shock proteins was induced in the D_TER strain with the highest levels of expression relative to the wild-type strain observed for *HSP12* (HPODL_03445, 2.6-fold induction), *HSP60* (HPODL_03254, 2.6-fold induction), *HSP70* (HPODL_02193, 2.7-fold induction) and highly expressed heat shock glycoprotein gene HPODL_00828 (2.4-fold induction). However, the antioxidant systems seem to be only partly affected by telomerase deficiency. Expression of peroxisomal catalase (HPODL_04626), and two out of four superoxide dismutase genes (HPODL_02458 and HPODL_01412) was up-regulated, while expression of other key enzymes, peroxiredoxins, the cytosolic thioredoxin and glutathione-based defense enzymes, did not change significantly or were slightly down-regulated in the D_TER strain (Additional file 2: Table S6).

### Carbohydrate and energy metabolism

The telomerase deficiency appeared to have a considerable impact on the cell metabolism. Genes assigned to the KEGG category “Carbohydrate Metabolism” were mostly induced in the D_TER strain (19% induced vs. 10% repressed), as well as “Energy Metabolism” genes (23% induced vs. 10% repressed).

Glycolysis is the central pathway for carbohydrate metabolism in yeast. All strains were grown in the presence of glucose, suggesting that the glycolytic enzymes operate in the direction of glucose fermentation. In the D_TER strain, expression of almost all glycolytic enzyme genes was downregulated (Additional file 2: Table S7). In the case of the pentose phosphate pathway, most of the genes involved in this pathway did not show significant changes in expression levels between the D_TER and wild-type strains (Additional file 2: Table S8). The only exception is the non-oxidative phase enzyme, transketolase (HPODL_04404), which was 2.6-fold up-regulated in the D_TER strain.

The expression of pyruvate metabolic enzymes shows multidirectional trends (Additional file 2: Table S7), while expression of pyruvate carboxylase was up-regulated almost two-fold, the level of pyruvate decarboxylase significantly drops (3.5-fold). Expression of pyruvate dehydrogenase was not significantly changed in the D_TER strain. Coordinated up-regulation of pyruvate carboxylase, converting pyruvate to oxaloacetate, and down-regulation of pyruvate decarboxylase that decarboxylate pyruvate to acetaldehyde and carbon dioxide, could serve an anaplerotic role supplying oxaloacetate for the tricarboxylic acid cycle when its intermediates are removed for different biosynthetic purposes. This process could be particularly important in senescent cells.

The tricarboxylic acid cycle genes were mostly unchanged (Additional file 2: Table S9). On the contrary, genes encoding mitochondrial cytochrome *c* oxidase and its accessory proteins were strongly down-regulated in the D_TER strain (Additional file 2: Table S10). Mitochondrial NADH dehydrogenase genes were induced (Additional file 2: Table S11). The expression of genes encoding multiple components of the F_1_F_0_ ATP synthetase shows different trends. The alpha subunit was up-regulated, subunits gamma and d were down-regulated, and expression of other subunits was not changed significantly (Additional file 2: Table S12). These observations differ from the previously reported data on the telomerase-deficient *S. cerevisiae* strain, where a marked up-regulation of genes encoding multiple components of the ATP synthetase and the electron transport chain was reported [10].

## Discussion

The genome-wide expression response to telomerase deficiency, the telomerase deletion response, have been previously reported in the case of deletion of telomerase RNA gene in *S. cerevisiae* [10]. We have studied another yeast species, *H. polymorpha*, and found quite different transcription response in a strain carrying a mutation in telomerase RNA gene. Analysis of telomerase-deficient senescing cells revealed a complex pattern of gene expression changes, some of them specific to telomerase loss and others resulting from the activation of the DNA damage response induced by critically short telomeres and cell cycle arrest. As in the case of deletion of telomerase RNA gene in *S. cerevisiae* [10], we observed some features similar to mammalian cells that have been senesced due to telomere shortening, such as activation of stress responses, inhibition of glycolysis, and down-regulation of mRNAs encoding the core histones [58]. In *S. cerevisiae*, telomere shortening resulted in down-regulation of Rap1, a central negative regulator of telomere length [10]. In *H. polymorpha*, two Rap proteins (Rap1A and Rap1B) were identified. Transcription of the first gene, *RAP1A*, was not changed significantly, while *RAP1B* was down-regulated. One can suggest that two proteins could play different roles associated with telomere shortening in *H. polymorpha*.

Telomerase RNA biogenesis in *Schizosaccharomyces pombe* involves sequential interaction with the Sm and Lsm complexes [59]. We have found that expression of the genes encoding the members of Sm and Lsm family proteins was mostly down-regulated. We have not previously detected putative Sm-site in telomerase RNA of *H. polymorpha* [16]. Down-regulation of all genes of this class can be explained by a possible novel regulatory role of these proteins for protein biogenesis machinery proposed by the splicing-independent interaction of Sm-proteins and mRNA [60]. Janssens and co-workers [61] mapped the proteome and transcriptome during the replicative lifespan of budding yeast and concluded that protein biogenesis machinery is a driver of replicative aging in this yeast.

It is currently unknown which proteins (Rap1, Rif1, CST complex or others) are involved in telomere maintenance in *H. polymorpha* [62]. We have found a difference in expression of *RAP1B* (HPODL_04303) and *RIF1* (HPODL_04218) genes for a strain with ever shorter telomere phenotype, suggesting that these proteins take part in the regulation of telomere length. Expression of genes encoding putative protein Cdc13 that is important for telomere length maintenance and transitions between the different steps of telomere elongation in *S. cerevisiae* [63, 64], and the main protein telomerase subunits Est2 and Est3, was not significantly changed.

Telomerase deficiency in *H. polymorpha* has a connection to autophagy. This is an expected result because the autophagic degradation declines with age and may be one of the reasons for the deformation of biological systems during aging [65]. Despite the fact that transcription of only a part of autophagy-related genes was changed, our data suggest that the regulation of the autophagy is perturbed upon telomerase loss.

Telomerase deficiency apparently activates DNA damage response due to inactivation of telomerase complex that protects telomeres. DNA damage-inducible changes in gene expression have been extensively studied in *S. cerevisiae* employing either treatment with chemical mutagens (e.g., methyl methanesulfonate) or ionizing radiation [66, 67]. In *S. cerevisiae*, these treatments induce so-called “DNA damage signature cluster” [67], including genes encoding the ribonucleotide reductase Rnr2 (small subunit), DNA repair proteins Rad51, and Rad54, cell-cycle checkpoint serine-threonine kinase Dun1, mitochondrial nuclease Din7, transcription factor Plm2, and two uncharacterized ORFs (*YER004W* and *YBR070C*). Five of these genes (*RNR2, RAD51, RAD54, DUN1, DIN7*) were upregulated in the mutant strain (Additional file 2: Table S5). The HPODL_01654 gene, encoding the large subunit of ribonucleotide reductase, was also strongly induced. Only a subset of DNA repair systems appeared to be up-regulated in senescent *H. polymorpha* cells: double-strand break repair via homologous recombination, non-homologous end joining pathway, and post-replication repair systems, while nucleotide excision repair, base excision repair, and mismatch repair systems were mostly unaffected.

In the senescent *S. cerevisiae* cells, expression of approximately 650 genes, called telomerase deletion response genes, changed two-fold or more relative to wild-type [10]. In particular, Nautiyal et al. [10] described 12 “telomerase deletion signature” genes reproducibly up-regulated in the course of telomere shortening but not under other conditions of stress and DNA damage: genes involved in ribosomal biogenesis and/or RNA processing (*EMG1, RPL37A, RRP43, BUD31*), cyclin-dependent kinase activating kinase *CAK1,* a putative cruciform DNA-binding protein *CRP1, YHR115C* containing a forkhead-associated (FHA) domain, glycosidase *CRH1*, a subunit of rapamycin-sensitive complex TORC1 involved in growth control *YHR186C* [68], integral membrane protein gene *YHR181W* and two genes with unknown functions, *YJL118W* and *YHT018C.* Five of these genes were not identified in *H. polymorpha,* and five others exhibit no significant changes of expression or moderate repression (Additional file 2: Table S13). Up-regulation in the D_TER strain was detected only for two genes, *YHR186C* (HPODL_00261), and *CRH1* (HPODL_03746). Up-regulation of HPODL_00261, encoding a subunit of TORC1 complex, seems to be clearly relevant to telomerase deficiency since TOR-depleted yeast cells display several physiological properties characteristic of starved or stressed cells, including a reduction of translation, an inhibition of ribosome biogenesis, specific metabolic changes and an induction of autophagy [69]. The strongest up-regulation in D_TER mutant of *H. polymorpha* was observed for a homolog of *CRH1* glycosidase, HPODL_03746. This enzyme belongs to the GH16 family transglycosylases involved in cell wall biosynthesis. Up-regulation of this gene may suggest altered cell wall composition in senescent *H. polymorpha* cells.

In *S. cerevisiae*, the set of telomerase deletion response genes included different functions, including stress response, protein synthesis and folding, cell wall maintenance, energy production, carbohydrate, and phosphate metabolism, RNA processing, and nucleotide synthesis [10]. However, among genes up-regulated two-fold or more in *S. cerevisiae* at senescence and found in *H. polymorpha* DL-1, only 19% (13 of 68) were also upregulated, while 12% (8 of 68) was downregulated. No correlation was observed in changes of expression of genes relevant to core metabolic pathways. The up-regulation of energy production genes, including components of the F_1_F_0_ ATP synthetase, the electron transport chain, tricarboxylic acid cycle genes, and ethanol utilization genes, suggested that senescing *S. cerevisiae* cells shifted from anaerobic to aerobic respiration [10]. Such a trend was not observed for *H. polymorpha*, in which down-regulation was observed for cytochrome *c* oxidase. A good correlation was observed for information processing and stress response functions, namely, repression of genes encoding ribosomal proteins and core histones, induction of genes involved in DNA damage response.

## Conclusions

In *H. polymorpha* cells, telomere shortening evoked transcriptional responses involving genes relevant to telomere structure and maintenance, DNA damage response, information processing and some metabolic pathways. Genes involved in DNA replication and repair, response to environmental stresses and intracellular traffic were up-regulated in senescent *H. polymorpha* cells, while down-regulation was observed for genes involved in transcription and translation, as well as core histones. Comparison of telomerase deletion transcription response in *S. cerevisiae* and *H. polymorpha* demonstrates that senescence makes a different impact on the main metabolic pathways of this yeast species but induces similar changes in processes related to nucleic acids metabolism and protein synthesis.

## Methods

### Strains preparation

*H. polymorpha* strain DL-1 (ATCC 26012) carrying the *LEU2* mutation was used as a control strain with intact telomerase (wild-type strain).

Construction of the D_TER strain was described previously [16]. After transformation of wild-type strain with the disruption constructs containing LEU2 for the HpTER knockout, selection was performed on complete minimal medium without leucine [16]. Several single colonies were inoculated into 2 ml of SD medium supplemented with leucine (0.67% YNB, 1% glucose, 20 mg/l leucine) to exclude influence on growth by activation of inserted LEU2. After growing overnight (passage 1), aliquots of the cultures were used for PCR verification of the hpTER gene replacement. Two of these D_TER cultures (clone #1 and clone #2 with the disrupted HpTER gene) were diluted to OD_600_ = 0.1 with 100 ml of SD medium supplemented with leucine and grown overnight again (passage 2). The two cultures were passaged three more times (passages 3, 4 and 5). At the end of each passage cell, viability and telomere length were analyzed. Telomere length was not measured after the passage 1, due to the low amount of cells. Ten milliliters of cells for RNA-seq analysis were harvested at OD_600_ ~ 1 during passages 2 and 3 for the clone #1 and passages 3 and 4 for the clone #2. Two colonies of the wild-type strain were passaged similarly, and two 10 ml aliquots (OD600 ~ 1) were harvested during passage 3 for RNA-seq. Cells were harvested by centrifugation (4000 rpm, 10 min, 4 °C) and taken up in AE-buffer (50 mM sodium acetate, 10 mM EDTA, pH 5.0).

### Cell viability assay

To analyze viability, samples from each culture were diluted to OD_600_ = 0.005 and 0.0005. Five microliters of the samples were plated onto YPD plates (1% yeast extract, 2% peptone, 2% glucose) and grown for 24 h. Numbers of viable colonies were normalized to the OD_600_. To determine the cell cycle distribution, cells during each passage (at OD_600_ ~ 1) (after mild sonication) were examined under a microscope (EVOS™ FL Cell Imaging System (Thermo Fisher Scientific) using 60× objective). Cells with buds less than one-half the size of mother cell were considered small-budded. More than 100 cells were counted for each passage.

### Southern blot analysis of telomere length

Telomere length was analyzed by Southern blot hybridization essentially as described previously [16]. Briefly, 40–60 μg of EcoRI-treated genomic DNA (DNA concentration was determined by UV absorbance at 260 nm) was separated on 1% agarose gel and transferred onto a Hybond N+ membrane (GE Healthcare Life Sciences). 5′-radiolabeled C4 oligonucleotide 5′-(CGCCACCC)_4_ was used as a probe to visualize telomere-containing restriction fragments.

### Transcriptome sequencing and analysis

The total RNA was extracted by a hot phenol method followed by purification using RNeasy Mini Kit (Qiagen). Six total RNA samples were used for transcriptome analysis, two from the wild-type strain and two from each of two independent D_TER strains. All of them were biological replicates (i.e., different cultures). mRNA library preparation was performed using an NEBNext® mRNA Library Prep Reagent Set for Illumina® according to the manufacturer’s instructions (New England BioLabs Inc., Ipswich, MA, USA). The libraries were sequenced using the Illumina HiSeq 2500 platform. About five million of 100-bp single end reads were generated for each sample. Illumina reads were mapped to the genes annotated in the *H. polymorpha* DL-1 genome (GenBank ID AEOI02000000) using Bowtie 2 [70], and the number of reads mapped to each particular gene was normalized using DESeq software [71]. Average numbers of mapped reads for four D_TER samples and two wild-type samples were compared to reveal up- or down-regulation of a particular gene. *P*-value was calculated to evaluate the significance of the differences between the expression levels in wild-type and D_TER strains.

### Analysis of apurinic/apyrimidinic endonuclease activity in whole cell extracts

The reaction mixtures (20 μl) contained 20 mM Hepes, pH 7.5, 100 mM NaCl, 5 mM MgCl_2_, 0.1 mg/ml BSA, 200 nM 5′-[^32^P]-labelled AP site-containing DNA (DNA-AP) and 0.15 mg/ml protein extracts. AP sites were generated immediately before experiments by treatment of the DNA duplex with *E. coli* uracil DNA glycosylase (5 U/pmol of the DNA duplex). The reaction mixtures were incubated at 37 °C for 30, 60 and 90 s. The AP endonuclease reactions were stopped by adding of 90% formamide, 10 mM EDTA, 0.1% bromophenol blue, and 0.1% xylene cyanol. The mixtures were heated at 90 °C for 3 min, and the products were separated by electrophoresis in 20% polyacrylamide gel (PAGE) containing 7 M urea. The gels were dried and subjected to phosphorimaging for quantification using Molecular Imager/Quantity One software (Bio-Rad, USA). Processing of AP site resulted in the appearance of ~15 nt band.

## Additional files

Additional file 1: Figure S1. Approximate distribution of cells throughout the cell cycle (for clone #2). **Figure S2.** Transcriptional landscape of the *H. polymorpha* genome. (PDF 157 kb)

Additional file 2: Supplementary tables presenting the data on differential expression of *H. polymorpha* DL-1 genes. **Table S1** Differential expression of *H. polymorpha* DL-1 genes classified into KEGG groups. **Table S2.** Expression levels of *H. polymorpha* DL-1 genes related to telomere maintenance. **Table S3.** Expression levels of *H. polymorpha* DL-1 genes relevant to autophagy. **Table S4.** Expression levels of *H. polymorpha* DL-1 genes relevant to cell architecture and intracellular traffic. **Table S5.** Expression levels of *H. polymorpha* DL-1 genes related to DNA damage checkpoint signaling, DNA replication and repair. **Table S6.** Expression levels of *H. polymorpha* DL-1 antioxidant system and heat shock genes. **Table S7.** Expression levels of *H. polymorpha* DL-1 genes involved in glycolysis, gluconeogensis and pyruvate metabolism. **Table S8.** Expression levels of *H. polymorpha* DL-1 pentose phosphate pathway genes. **Table S9.** Expression levels of *H. polymorpha* DL-1 tricarboxylic acids cycle genes. **Table S10.** Expression levels of *H. polymorpha* DL-1 genes encoding cytochrom *c* oxidase and related proteins. **Table S11.** Expression levels of *H. polymorpha* DL-1 genes encoding the NADH dehodrogenase subunits. **Table S12.** Expression levels of *H. polymorpha* DL-1 genes encoding the ATP synthase subunits. **Table S13.** Expression of homologs of telomerase deletion signature genes of *Saccharomyces cerevisiae* described in Nautiyal et al. (2002). (PDF 418 kb)

## Abbreviations

AP: Apurinic/apyrimidinic
ATG genes: Autophagy-related genes
BER: Base excision repair
BIR: Break induced replication
DDR: DNA damage response
DSB: Double strand break
EST: Ever shorter telomere
HR: Homologous recombination
MMR: Mismatch repair
NER: Nucleotide excision repair
NHEG: Non-homologous end joining
PRR: Post-replication repair
RTE: Recombinational telomere elongation
WT: Wild type
