# Supplementary material for: The genome-wide transcription response to telomerase deficiency in the thermotolerant yeast Hansenula polymorpha DL-1
Source: BMC Genomics. 2017 Jun 28;18:492. doi: 10.1186/s12864-017-3889-x (PMC5490237; doi:10.1186/s12864-017-3889-x)
Supplement: Supplementary file 1 — Approximate distribution of cells throughout the cell cycle (for clone #2). Figure S2. Transcriptional landscape of the H. polymorpha genome. (PDF 157 kb) [file 12864_2017_3889_MOESM1_ESM.pdf]

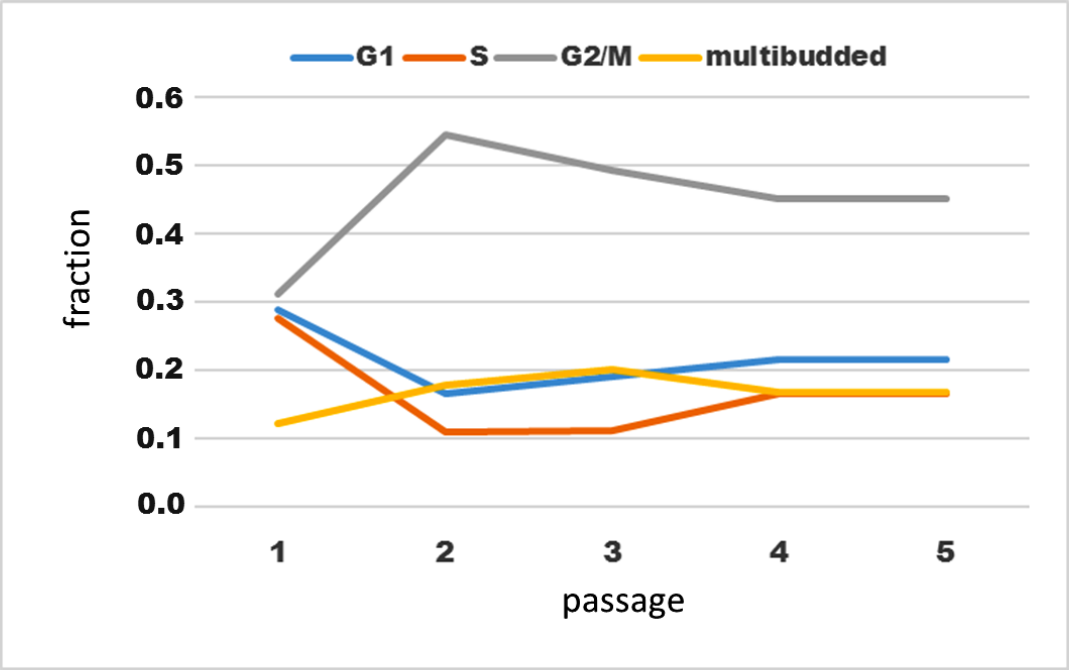

<u>Figure S1</u>. Approximate distribution of cells throughout the cell cycle (for clone#2).

Fraction of unbudded cells (G1) corresponding roughly to G1 phase, small-budded (S) to late G1 and S phases, and large-budded (G2/M) to G2/M phase and early G1 phase. (multibudded), - multibudded cells.

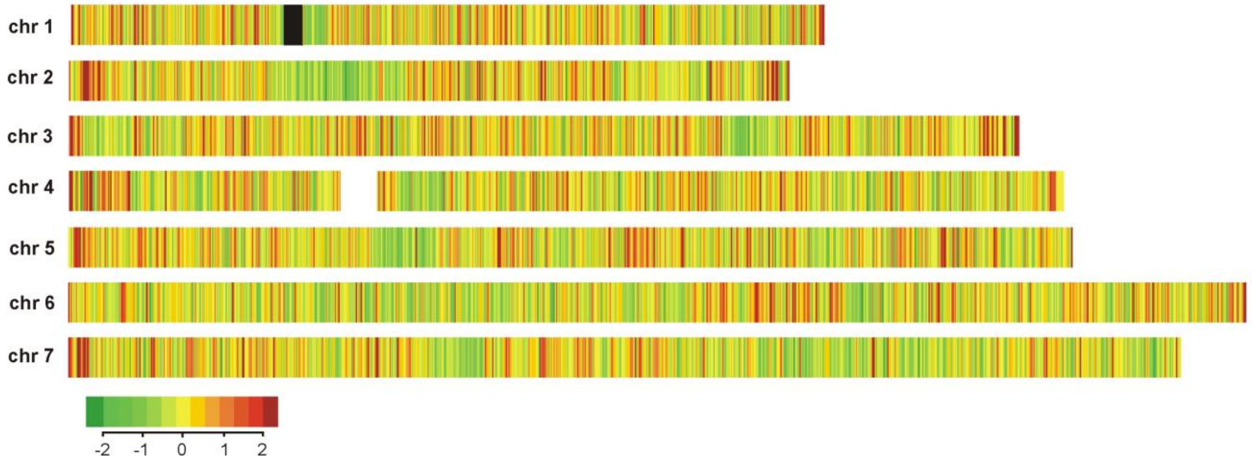

<u>Figure S2</u>. Transcriptional landscape of the *H. polymorpha* genome.

Heat map showing chromosomal distribution of transcribed genes in telomerase-deficient mutant strains coloured according to expression levels relative to wild type. Black box in chromosome 1 represents cluster of genes encoding ribosomal RNA. The colour code for normalized transcript count is shown in the bar below (Log2 fold change).
