# Supplementary material for: The genome-wide transcription response to telomerase deficiency in the thermotolerant yeast Hansenula polymorpha DL-1
Source: BMC Genomics. 2017 Jun 28;18:492. doi: 10.1186/s12864-017-3889-x (PMC5490237; doi:10.1186/s12864-017-3889-x)
Supplement: Supplementary file 2 — Supplementary tables presenting the data on differential expression of H. polymorpha DL-1 genes. Table S1 Differential expression of H. polymorpha DL-1 genes classified into KEGG groups. Table S2. Expression levels of H. polymorpha DL-1 genes related to telomere maintenance. Table S3. Expression levels of H. polymorpha DL-1 genes relevant to autophagy. Table S4. Expression levels of H. polymorpha DL-1 genes relevant to cell architecture and intracellular traffic. Table S5. Expression levels of H. polymorpha DL-1 genes related to DNA damage checkpoint signaling, DNA replication and repair. Table S6. Expression levels of H. polymorpha DL-1 antioxidant system and heat shock genes. Table S7. Expression levels of H. polymorpha DL-1 genes involved in glycolysis, gluconeogensis and pyruvate metabolism. Table S8. Expression levels of H. polymorpha DL-1 pentose phosphate pathway genes. Table S9. Expression levels of H. polymorpha DL-1 tricarboxylic acids cycle genes. Table S10. Expression levels of H. polymorpha DL-1 genes encoding cytochrom c oxidase and related proteins. Table S11. Expression levels of H. polymorpha DL-1 genes encoding the NADH dehodrogenase subunits. Table S12. Expression levels of H. polymorpha DL-1 genes encoding the ATP synthase subunits. Table S13. Expression of homologs of telomerase deletion signature genes of Saccharomyces cerevisiae described in Nautiyal et al. (2002). (PDF 418 kb) [file 12864_2017_3889_MOESM2_ESM.pdf]

Table S1. Differential expression of *H. polymorpha* DL-1 genes classified into KEGG groups

**KEGG groups**	**Total number of genes**	**Genes upregulated in D_TER strain (x>2)**	**No significant changes in expression (2>x>0.5)**	**Genes downregulated in D_TER strain (x<0.5)**
**Metabolism**				
1. Carbohydrate metabolism	**188**	36	134	18
2. Energy metabolism	**125**	29	84	12
3. Lipid metabolism	**113**	13	90	10
4. Nucleotide metabolism	**114**	12	99	3
5. Amino acid metabolism	**183**	31	136	16
6. Glycan biosynthesis and metabolism	**68**	8	58	2
7. Metabolism of cofactors and vitamins	**115**	8	95	12
8. Biosynthesis of other secondary metabolites	**29**	8	18	3
9. Xenobiotics biodegradation and metabolism	**45**	15	28	2
**Genetic Information Processing**				
10. Transcription	**125**	4	110	11
11. Translation	**294**	5	267	22
12. Folding, sorting and degradation	**211**	19	175	17
13. Replication and repair	**85**	16	68	1
**Environmental Information Processing**				
14. Signal transduction	**146**	20	111	15
**Cellular Processes**				
15. Transport and catabolism	**123**	14	100	9
16. Cell growth and death	**140**	26	106	8

Table S2. Expression levels of *H. polymorpha* DL-1 genes related to telomere maintenance

In this and subsequent table the relative expression levels (normalized coverage) of significantly upregulated genes ($P < 0.05$) are shown in red, and ones upregulated more than 2-fold relative to WT are shown in **red and boldface**. The expression levels of significantly downregulated genes ($P < 0.05$) are shown in blue, and ones downregulated more than 2-fold are shown in **blue and boldface.** The normalized coverage data are averages of four D_TER samples and two wild type (WT) samples.

**Function**	**Gene ID**	**Normalized coverage**			
		**WT**	**D_TER**	**Fold change**	***P* value**
telomerase RNA		65	5	**0,07**	2 e-06
hypothetical protein Est1	HPODL_01459	508	482	0,95	0,71
telomerase reverse transcriptase Est2	HPODL_01277	193	145	0,75	0,07
hypothetical protein Est3	HPODL_02192	41	58	1,44	0,09
hypothetical protein Ku70	HPODL_02050	63	95	1,52	0,03
hypothetical protein Ku80	HPODL_01499	68	116	1,71	0,00
hypothetical protein Cdc13	HPODL_00415	79	110	1,38	0,08
Protein TBF1	HPODL_03795	212	161	0,76	0,07
Sm protein SMB	HPODL_00463	357	212	0,59	0,04
small nuclear ribonucleoprotein Sm D1	HPODL_01396	126	79	0,63	0,01
Small nuclear ribonucleoprotein Sm D2	HPODL_00571	127	89	0,70	0,04
Small nuclear ribonucleoprotein E	HPODL_01477	64	40	0,63	0,02
Small nuclear ribonucleoprotein F	HPODL_03956	182	93	0,51	0,04
Small nuclear ribonucleoprotein G	HPODL_02984	258	149	0,58	0,00
LSm protein LSM2	HPODL_05263	1031	378	**0,37**	6 e-08
Sm-like protein LSm4	HPODL_00774	535	211	**0,39**	0,00
Sm-like protein LSm5	HPODL_03344	461	189	**0,41**	1 e-06
Sm-like protein LSm1	HPODL_03359	324	184	0,57	9 e-05
LSm protein LSM3	HPODL_05140	756	216	**0,29**	0,00
LSm protein LSM7	HPODL_04033	628	397	0,63	0,00
Protein PXR1	HPODL_01680	165	112	0,68	0,26
DNA-binding protein RAP1A	HPODL_03159	553	458	0,83	0,17
DNA-binding protein RAP1B	HPODL_04303	303	138	**0,46**	1e-07
Hypothetical protein Stn1	HPODL_03833	107	172	1,60	0,01
Hypothetical protein Rif1	HPODL_04218	364	706	1,94	6 e-06

Table S3. Expression levels of *H. polymorpha* DL-1 genes relevant to autophagy

Function	Gene ID	Normalized coverage			
		WT	D_TER	Fold change	*P* value
Autophagy-related protein 21	HPODL_01040	24	40	1,67	0,05
Autophagy-related protein 22	HPODL_03790	121	139	1,15	0,44
Autophagy-related protein 13	HPODL_03959	138	268	1,94	4 e-05
Autophagy-related protein 25	HPODL_01515	78	81	1,04	0,86
Autophagy-related protein 11	HPODL_05134	74	122	1,65	0,01
Putative lipase ATG15	HPODL_03503	77	134	1,75	0,00
Serine/threonine-protein kinase ATG1	HPODL_04379	256	271	1,06	0,77
cysteine protease ATG4	HPODL_04394	77	111	1,45	0,08
Subunit of TORC1, a rapamycin-sensitive complex involved in growth control	HPODL_00261	145	277	1,90	5 e-05
Target of rapamycin complex subunit LST8	HPODL_04064	151	114	0,75	0,08
cAMP-dependent protein kinase type 2	HPODL_04656	78	206	**2,63**	3 e-08
Serine/threonine-protein kinase SCH9	HPODL_03332	238	360	1,51	0,01
serine/threonine-protein kinase RIM15	HPODL_04619	259	468	1,81	7 e-05
Putative lipase ATG15	HPODL_03503	77	134	1,75	0,00

Table S4. Expression levels of *H. polymorpha* DL-1 genes relevant to cell architecture and intracellular traffic.

Function	Gene ID	Normalized coverage			
		WT	D_TER	Fold change	*P* value
histone acetylase	HPODL_01348	89	87	0,98	0,88
Histone acetyltransferase catalytic subunit of NuA3 complex	HPODL_00848	80	77	0,96	0,83
Histone acetyltransferase ESA1	HPODL_01019	105	160	1,52	0,01
Histone acetyltransferase GCN5	HPODL_03013	339	165	0,49	8 e-07
Histone acetyltransferase RTT109	HPODL_01542	69	69	1,00	0,98
Histone acetyltransferase SAGA associated factor SGF29	HPODL_00193	223	139	0,62	0,00
histone acetyltransferase Spt10	HPODL_04452	452	209	**0,46**	5 e-08
Histone acetyltransferase type B catalytic subunit	HPODL_02220	62	71	1,15	0,51
Histone acetyltransferase type B subunit 2	HPODL_02956	232	159	0,69	0,05
Histone acetyltransferase type B subunit 2	HPODL_03084	87	106	1,22	0,29
Histone chaperone ASF1	HPODL_04174	96	148	1,54	0,01
Histone deacetylase complex subunit CTI6	HPODL_01866	66	94	1,42	0,08
Histone deacetylase HDA1	HPODL_04853	269	243	0,90	0,48
histone deacetylase HOS2	HPODL_04199	29	56	1,90	0,02
Histone deacetylase RPD3	HPODL_01828	370	395	1,07	0,63
Histone demethylase JHD2	HPODL_01805	576	348	0,60	0,01
histone H2A	HPODL_00354	4456	2690	0,60	0,01
Histone H2A.Z	HPODL_03574	254	222	0,88	0,37
histone H2B	HPODL_00353	5035	2764	0,55	0,00
histone H2B	HPODL_00515	3808	2003	0,53	2 e-05
histone H3	HPODL_01931	311	110	**0,36**	1 e-08
Histone H3.1/H3.2	HPODL_01324	4124	2337	0,57	0,03
Histone H3.1/H3.2	HPODL_01891	3987	2216	0,56	0,04
Histone H4	HPODL_01325	13771	5365	**0,39**	5 e-06
Histone H4	HPODL_01892	6519	2646	**0,41**	4 e-05
histone-binding protein RBBP4	HPODL_03248	206	317	1,54	0,05
histone-lysine N-methyltransferase SETD1	HPODL_02902	283	236	0,83	0,29
histone-lysine N-methyltransferase SETD2	HPODL_04982	151	162	1,08	0,65
histone-lysine N-methyltransferase, H3 lysine-79 specific	HPODL_00709	200	211	1,06	0,75
kinesin family protein (KlpA), putative	HPODL_00141	104	116	1,12	0,54
kinesin heavy chain	HPODL_00616	91	132	1,45	0,03

Kinesin-like protein bimC	HPODL_00992	161	146	0,91	0,57
Kinesin-like protein K39	HPODL_03466	363	536	1,47	0,01
Kinesin-like protein KIF13B	HPODL_02966	226	163	0,72	0,03
Kinesin-like protein KIP3	HPODL_03526	122	159	1,30	0,11
Kinesin-related motor protein involved in mitotic spindle positioning	HPODL_02234	118	170	1,45	0,03
kinetochor protein Mis14/NSL1	HPODL_01652	146	157	1,08	0,87
Kinetochore protein mis13	HPODL_03681	296	210	0,71	0,02
Kinetochore protein NDC80	HPODL_00462	662	694	1,05	0,75
kinetochore protein NUF2	HPODL_01577	194	244	1,26	0,13
Kinetochore-associated protein MTW1	HPODL_00238	88	44	0,50	0,00
Kinetochore-associated protein NNF1	HPODL_03927	82	83	1,02	0,95
Dynein heavy chain, cytoplasmic, cytosolic	HPODL_03488	291	787	**2,71**	3 e-11
Tubulin alpha chain	HPODL_04315	1379	2494	1,81	2 e-05
Tubulin beta chain	HPODL_02853	988	2000	**2,02**	4 e-07
Tubulin gamma chain	HPODL_02310	157	137	0,87	0,39
Tubulin-tyrosine ligase	HPODL_04942	1541	613	**0,40**	1 e-12

Table S5. Expression levels of *H. polymorpha* DL-1 genes related to DNA damage checkpoint signaling, DNA replication and repair.

Function	Gene ID	Normalized coverage			
		WT	D_TER	Fold change	*P* value
**DNA damage signature cluster**					
Ribonucleoside-diphosphate reductase small chain 1 (RNR2)	HPODL_03249	459	3284	**7,15**	7 e-37
Cell-cycle checkpoint serine-threonine kinase DUN1	HPODL_01729	99	208	**2,10**	1 e-05
nuclease DIN7	HPODL_02487	108	193	1,78	0,00
transcription factor PLM2	HPODL_00413	217	196	0,90	0,50
Ribonucleoside-diphosphate reductase large chain 1	HPODL_01654	360	1570	**4,36**	5 e-12
DNA repair protein Rad51	HPODL_03021	138	475	**3,45**	2 e-10
DNA repair protein Rad54	HPODL_04466	151	342	**2,26**	2 e-07
**DNA damage signaling checkpoint proteins**					
hypothetical protein Rad17	HPODL_00305	105	176	1,67	0,00
hypothetical protein Ddc1	HPODL_00259	84	109	1,29	0,15
checkpoint protein Rad24	HPODL_04046	126	183	1,45	0,02
hypothetical protein Mec1	HPODL_03100	378	355	0,94	0,77
hypothetical protein Ddc2	HPODL_00412	665	299	**0,45**	0,01
transcription-associated protein (TEL1)	HPODL_00447	515	864	1,68	0,00
hypothetical protein Rad9	HPODL_02495	165	214	1,29	0,11
mediator of replication checkpoint protein 1 (Mrc1)	HPODL_00404	161	204	1,27	0,15
DNA damage response protein kinase Rad53	HPODL_03533	220	207	0,94	0,67
Serine/threonine kinase and DNA damage checkpoint effector (Chk1)	HPODL_02468	190	180	0,94	0,70
cell-cycle checkpoint serine-threonine kinase (Dun1)	HPODL_01729	99	208	**2,10**	1 e-05
**DNA replication**					
DNA polymerase alpha catalytic subunit A (Pol 1)	HPODL_02887	438	417	0,95	0,75
B subunit of DNA polymerase alpha-primase complex (Pol 12)	HPODL_04277	99	115	1,16	0,39
DNA primase large subunit (Pri1)	HPODL_03502	195	152	0,78	0,12
DNA primase small subunit (Pri2)	HPODL_00382	172	137	0,80	0,46
DNA polymerase delta catalytic subunit (Pol3)	HPODL_04232	122	293	**2,41**	7 e-08
DNA polymerase delta subunit (Pol31)	HPODL_01067	102	139	1,36	0,07

hypothetical protein Pol32	HPODL_02467	128	240	1,87	0,00
DNA polymerase epsilon catalytic subunit A (Pol2)	HPODL_02497	178	264	1,48	0,01
DNA polymerase epsilon subunit 2 (DPB2)	HPODL_01201	148	186	1,25	0,16
DNA polymerase epsilon subunit C (DPB3)	HPODL_03599	94	88	0,94	0,70
DNA polymerase epsilon subunit 3 (DPB4)	HPODL_03329	205	173	0,84	0,45
DNA replication licensing factor MCM2	HPODL_03181	301	342	1,14	0,38
DNA replication licensing factor MCM4	HPODL_00647	135	252	1,87	0,00
DNA replication licensing factor mcm6	HPODL_04666	143	167	1,17	0,33
DNA replication licensing factor mcm3	HPODL_04438	239	416	1,74	0,00
DNA replication licensing factor mcm5	HPODL_03794	121	109	0,90	0,56
DNA replication licensing factor MCM7	HPODL_01197	443	576	1,30	0,10
Proliferating cell nuclear antigen (PCNA)	HPODL_04911	239	297	1,24	0,15
Replication factor C subunit 1 (RFC1)	HPODL_01204	186	349	1,88	4 e-05
Replication factor C subunit 2 (RFC2)	HPODL_03600	179	172	0,96	0,83
Replication factor C subunit 3 (RFC3)	HPODL_02081	47	72	1,53	0,04
Replication factor C subunit 4 (RFC4)	HPODL_01108	314	282	0,90	0,48
Subunit of heterotrimeric Replication Protein A (RPA1)	HPODL_00221	264	866	**3,28**	1 e-14
Replication factor A protein 2 (RPA2)	HPODL_03073	428	462	1,08	0,59
Single-stranded DNA-binding protein (RPA3)	HPODL_02102	592	420	0,71	0,01
ribonuclease HI (RNH1)	HPODL_01971	103	91	0,88	0,47
Flap endonuclease 1 (Rad27)	HPODL_01136	210	170	0,81	0,18
DNA ligase 1 (Cdc9)	HPODL_04991	231	263	1,14	0,39
**Base excision repair (BER)**					
Uracil-DNA glycosylase (Ung)	HPODL_00742	99	126	1,27	0,18
DNA-3-methyladenine glycosylase II (Mag1)	HPODL_03343	247	190	0,77	0,08
N-glycosylase/DNA lyase (Ogg1)	HPODL_03449	108	111	1,03	0,88
DNA-(apurinic/apyrimidinic site) lyase 1 (Apn1)	HPODL_01629	51	114	**2,22**	4 e-05
APN2 Class II abasic (AP) endonuclease (Apn2)	HPODL_04785	33	60	1,84	0,01
Polynucleotide kinase 3'	HPODL_01529	120	115	0,96	0,80

phosphatase (Tpp1)					
**Nucleotide excision repair (NER)**					
DNA repair protein Rad4	HPODL_01585	72	160	**2,21**	1 e-05
Protein with ubiquitin-like N terminus (Rad23)	HPODL_00558	300	426	1,42	0,02
hypothetical protein, Rad33	HPODL_03234	128	106	0,83	0,27
Protein that recognizes and binds damaged DNA in an ATP-dependent manner, Rad7	HPODL_00556	143	163	1,14	0,60
DNA repair protein Rad16	HPODL_02433	93	198	**2,12**	1 e-05
DNA repair like-protein Rad1	HPODL_00021	67	103	1,54	0,02
DNA excision repair protein ERCC-1, Rad10	HPODL_00169	101	75	0,74	0,10
single-stranded DNA endonuclease, Rad2	HPODL_03738	204	207	1,02	0,96
DNA repair protein Rad14	HPODL_03131	132	151	1,15	0,41
DNA repair helicase RAD25	HPODL_00461	250	266	1,07	0,68
DNA repair helicase RAD3	HPODL_01089	152	194	1,28	0,12
nucleotide excision repair factor TFIIH, subunit TFB1	HPODL_01026	63	111	1,77	0,00
Suppressor of stem-loop protein 1, SSL1	HPODL_03706	107	128	1,20	0,31
Transcription factor Tfb2	HPODL_04144	372	183	**0,49**	8 e-07
nucleotide excision repair factor TFIIH, subunit TFB4	HPODL_01646	330	205	0,62	0,00
hypothetical protein, Tfb5	HPODL_05230	29	21	0,73	0,26
DNA excision repair protein ERCC-6, RAD26	HPODL_04651	190	129	0,68	0,01
nucleotide excision repair factor TFIIH, subunit TFB3	HPODL_02425	107	93	0,87	0,42
hypothetical protein, TFIIS	HPODL_05118	238	140	0,59	0,00
hypothetical protein, RNA polymerase II transcription factor B subunit 3	HPODL_02181	31	73	2,33	0,00
**Mismatch repair (MMR)**					
Protein required for mismatch repair (MLH1)	HPODL_05041	106	104	0,98	0,90
DNA mismatch repair protein MSH6	HPODL_04045	449	689	1,53	0,00
DNA mismatch repair protein MSH2	HPODL_02329	319	321	1,01	1,00
DNA mismatch repair protein PMS1	HPODL_03630	23	46	**2,03**	0,00
DNA mismatch repair protein mutL (MLH3)	HPODL_00567	113	172	1,51	0,03
exonuclease 1, EXO1	HPODL_02487	108	193	1,78	0,00
Mismatch repair protein (MSH5)	HPODL_04591	165	196	1,18	0,28
**Double strand break repair via Homologous**					

**Recombination (HR)**					
Subunit of a complex with Rad50p and Xrs2p (MRX complex), MRE11	HPODL_02469	89	93	1,04	0,82
DNA repair protein RAD50	HPODL_02842	165	282	1,71	0,00
DNA repair protein rhp51 (RAD51)	HPODL_03021	138	475	**3,45**	2 e-10
DNA repair and recombination protein rad22 (RAD52)	HPODL_04607	56	146	**2,58**	4 e-07
DNA repair protein rhp54 RAD54	HPODL_04466	151	342	**2,26**	2 e-07
DNA repair protein RAD59	HPODL_04496	142	150	1,05	0,80
Structure-specific endonuclease subunit SLX1	HPODL_01576	59	146	**2,49**	1 e-06
Crossover junction endonuclease MUS81	HPODL_04818	15	89	**5,87**	6 e-08
Bloom syndrome protein SGS1	HPODL_04257	127	290	2,28	3 e-07
DNA topoisomerase 3 (TOP3)	HPODL_01455	95	179	1,89	0,00
DNA-dependent ATPase MGS1	HPODL_01063	78	96	1,24	0,23
**Non-homologous end joining (NHEJ)**					
hypothetical protein Ku70	HPODL_02050	63	95	1,52	0,03
ATP-dependent DNA helicase 2 subunit 2 (Ku80)	HPODL_01499	68	116	1,71	0,00
hypothetical protein, Lig4	HPODL_04963	72	107	1,49	0,03
**Post-replication repair (PRR)**					
E3 ubiquitin-protein ligase RAD18	HPODL_04934	58	122	**2,12**	7 e-05
Catalytic subunit of DNA polymerase zeta (REV3)	HPODL_03134	142	206	1,45	0,02
DNA polymerase eta subunit (RAD30)	HPODL_00454	43	110	**2,58**	2 e-06
DNA repair protein REV1	HPODL_02716	65	82	1,26	0,22

Table S6. Expression levels of *H. polymorpha* DL-1 antioxidant system and heat shock genes

Function	Gene ID	Normalized coverage			
		WT	D_TER	Fold change	*P* value
**Antioxidant system**					
Catalase	HPODL_04626	678	1334	1,97	1 e-06
Superoxide dismutase (Mn)	HPODL_01414	463	263	0,57	7 e-05
	HPODL_02458	251	1065	**4,25**	6 e-21
	HPODL_03894	522	506	0,97	0,82
Superoxide dismutase (Cu-Zn)	HPODL_01412	1096	2506	**2,29**	5 e-09
Peroxiredoxin	HPODL_03023	1802	2367	1,31	0,05
	HPODL_00398	1175	617	0,53	1 e-06
	HPODL_02878	11691	7083	0,61	6 e-05
	HPODL_03404	62	60	0,96	0,83
	HPODL_00527	1084	1291	1,19	0,21
	HPODL_03659	501	455	0,91	0,47
	HPODL_00254	181	185	1,03	0,88
Thioredoxin	HPODL_03852	236	206	0,87	0,34
	HPODL_01485	2103	1377	0,65	0,01
Thioredoxin reductase	HPODL_04814	358	523	1,46	0,01
Glutaredoxin	HPODL_04165	1302	893	0,69	0,00
	HPODL_02727	234	141	0,60	0,13
Glutathione S-transferase	HPODL_02388	796	732	0,92	0,51
	HPODL_03063	353	385	1,09	0,55
Glutathione reductase	HPODL_01177	553	770	1,39	0,02
Glutathione peroxidase	HPODL_03940	1416	831	0,59	0,02
Gamma-glutamylcysteine synthetase	HPODL_02265	349	400	1,14	0,35
Glutathione synthetase	HPODL_00931	180	270	1,50	0,01
**Heat shock factors**					
Heat shock transcription factor	HPODL_00106	951	892	0,94	0,63
Heat shock glycoprotein	HPODL_00828	5086	12133	**2,39**	2 e-06
Heat shock protein ClpB	HPODL_02027	672	417	0,62	0,02
Heat shock protein 12	HPODL_03445	386	996	**2,58**	1 e-10
Heat shock protein 26	HPODL_03869	160	51	**0,32**	0,00
Heat shock protein 42	HPODL_01889	160	112	0,70	0,16
Heat shock protein 60	HPODL_03254	722	1858	**2,57**	4 e-11
Heat shock protein 70 1	HPODL_02450	108	354	**3,29**	5 e-07
Heat shock protein 70 2	HPODL_02193	1925	5150	**2,67**	3 e-12
Heat shock protein 78	HPODL_03269	214	385	1,80	0,00
Heat shock protein Hsp88	HPODL_03970	2130	1805	0,85	0,23
Heat shock protein SSB1	HPODL_02202	3570	3761	1,05	0,68
Heat shock protein SSC1, mitochondrial	HPODL_04714	484	415	0,86	0,35
Heat shock protein SSC1, mitochondrial	HPODL_01106	2305	2627	1,14	0,45
Heat shock protein STI1	HPODL_00085	320	586	1,83	4 e-05
Hsp70 nucleotide exchange factor FES1	HPODL_02785	160	225	1,41	0,03

Hsp70/Hsp90 co-chaperone CNS1	HPODL_02744	108	86	0,80	0,19
Hsp90 co-chaperone AHA1	HPODL_02459	206	399	1,94	2 e-05
Hsp90 co-chaperone Cdc37	HPODL_03139	382	376	0,98	0,90
Cell wall mannoprotein HSP150	HPODL_01142	2077	2414	1,16	0,27

Table S7. Expression levels of *H. polymorpha* DL-1 genes involved in glycolysis, gluconeogensis and pyruvate metabolism

**Function**	**Gene ID**	**Normalized coverage**			
		**WT**	**D_TER**	**Fold change**	***P* value**
Glucokinase	HPODL_04126	7445	3278	**0,44**	0,00
Hexokinase	HPODL_04936	9328	2640	**0,28**	8 e-05
	HPODL_04428	25	64	**2,58**	5 e-05
Glucose-6-phosphate isomerase	HPODL_01256	4416	2446	0,55	0,02
Phosphofructokinase	HPODL_00776	4218	1616	**0,38**	3 e-09
	HPODL_01506	2160	1206	0,56	0,00
Fructose-bisphosphate aldolase	HPODL_03669	11580	5084	**0,44**	0,00
Triosephosphate isomerase	HPODL_03187	4111	2231	0,54	7 e-06
Glyceraldehyde-3-phosphate dehydrogenase	HPODL_04957	35867	16312	**0,45**	2 e-05
Phosphoglycerate kinase	HPODL_04829	5655	3110	0,55	0,01
Phosphoglycerate mutase	HPODL_01332	9108	4266	**0,47**	0,00
	HPODL_02910	185	120	0,65	0,01
	HPODL_00350	140	84	0,60	0,01
Enolase	HPODL_04582	12550	6400	0,51	3 e-05
Pyruvate kinase	HPODL_01513	5018	3260	0,65	0,00
Pyruvate carboxylase	HPODL_03803	1125	2105	1,87	6 e-06
Phosphoenolpyruvate carboxykinase	HPODL_00509	370	449	1,22	0,56
Fructose-1,6-bisphosphatase	HPODL_03321	422	484	1,15	0,47
Pyruvate decarboxylase	HPODL_04375	15271	4378	**0,29**	2 e-12
Pyruvate dehydrogenase (subunit alpha)	HPODL_00436	1640	1303	0,79	0,08
Pyruvate dehydrogenase (subunit beta)	HPODL_01422	2762	1923	0,70	0,01

Table S8. Expression levels of *H. polymorpha* DL-1 pentose phosphate pathway genes

Function	Gene ID	Normalized coverage			
		WT	D_TER	Fold change	*P* value
Glucose-6-phosphate dehydrogenase	HPODL_03864	723	1021	1,41	0,01
6-phosphogluconolactonase	HPODL_03047	275	283	1,03	0,88
	HPODL_01158	782	572	0,73	0,02
6-phosphogluconate dehydrogenase	HPODL_02085	3274	4746	1,45	0,01
Ribose-5-phosphate isomerase	HPODL_02541	413	286	0,69	0,04
Ribulose-phosphate 3-epimerase	HPODL_03355	313	355	1,14	0,50
Transketolase	HPODL_04404	1785	4550	**2,55**	3 e-11
	HPODL_02162	34	74	**2,19**	0,00
Transaldolase	HPODL_02007	5070	3433	0,68	0,00
	HPODL_02415	8	17	2,30	0,03
	HPODL_02008	246	135	0,55	8 e-05

Table S9. Expression levels of *H. polymorpha* DL-1 tricarboxylic acids cycle genes

Function	Gene ID	Normalized coverage			
		WT	D_TER	Fold change	*P* value
Citrate synthase, mitochondrial	HPODL_04359	1501	2628	1,75	4 e-05
Homoaconitase, mitochondrial	HPODL_02077	327	474	1,45	0,01
Isocitrate dehydrogenase [NADP], mitochondrial	HPODL_03527	1142	1107	0,97	0,80
Isocitrate dehydrogenase [NAD] subunit 1, mitochondrial	HPODL_01696	4628	3124	0,68	0,00
Isocitrate dehydrogenase [NAD] subunit 2, mitochondrial	HPODL_03428	1113	1735	1,56	0,00
2-oxoglutarate dehydrogenase, mitochondrial	HPODL_02153	673	2156	**3,20**	3 e-13
succinyl-CoA ligase subunit alpha, mitochondrial	HPODL_05098	1327	1623	1,22	0,15
succinyl-CoA ligase subunit beta, mitochondrial	HPODL_02040	1713	2084	1,22	0,15
Membrane anchor subunit of succinate dehydrogenase (Sdh1p, Sdh2p, Sdh3p, Sdh4p)	HPODL_02356	671	763	1,14	0,48
Membrane anchor subunit of succinate dehydrogenase (Sdh1p, Sdh2p, Sdh3p, Sdh4p)	HPODL_04163	125	81	0,65	0,01
Succinate dehydrogenase [ubiquinone] iron-sulfur subunit, mitochondrial	HPODL_04234	742	951	1,28	0,13
succinate dehydrogenase cytochrome B subunit, mitochondrial precursor	HPODL_01634	660	813	1,23	0,20
Succinate dehydrogenase [ubiquinone] flavoprotein subunit, mitochondrial	HPODL_02419	803	1725	**2,15**	5 e-08
Fumarate hydratase, mitochondrial	HPODL_00724	761	1389	1,82	2 e-05
Malate dehydrogenase, mitochondrial	HPODL_00710	988	3099	**3,14**	1 e-12
NAD-dependent malic enzyme, mitochondrial	HPODL_03986	1774	1713	0,97	0,85

Table S10. Expression levels of *H. polymorpha* DL-1 genes encoding cytochrom *c* oxidase and related proteins.

**Function**	**Gene ID**	**Normalized coverage**			
		**WT**	**D_TER**	**Fold change**	***P* value**
Cytochrome c	HPODL_04845	4242	2397	0,56	8 e-06
Cytochrome c oxidase assembly factor COX23	HPODL_04099	198	92	**0,46**	0,02
Cytochrome c oxidase assembly protein COX15	HPODL_04345	1141	568	0,50	6 e-06
Cytochrome c oxidase assembly protein COX11	HPODL_01149	308	212	0,69	0,01
Subunit IV of cytochrome c oxidase	HPODL_02096	7281	3931	0,54	1 e-06
Subunit Va of cytochrome c oxidase	HPODL_01334	2517	1647	0,65	0,01
Subunit Vb of cytochrome c oxidase	HPODL_01289	2380	1186	**0,50**	7 e-08
Subunit VIa of cytochrome c oxidase	HPODL_01292	3037	1830	0,60	9 e-05
Subunit VIb of cytochrome c oxidase	HPODL_03413	1928	1085	0,56	1 e-05
Subunit VIIc of cytochrome c oxidase	HPODL_05272	4939	1959	**0,40**	0,00

Table S11. Expression levels of *H. polymorpha* DL-1 genes encoding the NADH dehodrogenase subunits

Function	Gene ID	Normalized coverage			
		WT	D_TER	Fold change	*P* value
NADH dehydrogenase subunit I	HPODL_02101	61	196	**3,22**	1 e-10
NADH dehydrogenase subunit F	HPODL_04625	39	340	**8,68**	1 e-29
NADH-ubiquinone oxidoreductase subunit 10	HPODL_04828	70	106	1,51	0,10
NADH dehydrogenase (ubiquinone) 1 alpha subcomplex 5	HPODL_00771	200	197	0,98	0,89
NADH dehydrogenase subunit C	HPODL_03393	37	169	**4,59**	2 e-14
NADH dehydrogenase subunit G	HPODL_03689	109	342	**3,14**	4 e-12
NADH dehydrogenase 1 alpha subcomplex 8	HPODL_04116	98	232	**2,36**	3 e-07
NADH dehydrogenase (ubiquinone) Fe-S protein 6	HPODL_01160	66	146	**2,23**	1 e-05
NADH dehydrogenase subunit E	HPODL_01287	53	114	**2,17**	6 e-05
NADH dehydrogenase subunit D	HPODL_01297	44	296	**6,80**	2 e-24
NADH dehydrogenase subunit B	HPODL_02758	22	165	**7,35**	3 e-20
NADH dehydrogenase (ubiquinone) Fe-S protein 4	HPODL_02906	32	51	1,59	0,05

Table S12. Expression levels of *H. polymorpha* DL-1 genes encoding the ATP synthase subunits.

Function	Gene ID	Normalized coverage			
		WT	D_TER	Fold change	*P* value
F0F1-type ATP synthase subunit alpha, mitochondrial	HPODL_02577	4813	8031	1,67	0,00
F0F1-type ATP synthase subunit beta, mitochondrial	HPODL_01806	8349	9167	1,10	0,47
F0F1-type ATP synthase subunit gamma, mitochondrial	HPODL_00669	8348	3853	**0,46**	1 e-09
F0F1-type ATP synthase, subunit delta, mitochondrial	HPODL_01793	1228	1246	1,01	0,92
F0F1-type ATP synthase subunit d, mitochondrial	HPODL_02723	3598	2350	0,65	0,01
F0F1-type ATP synthase subunit b, mitochondrial	HPODL_02801	2251	2347	1,04	0,75
F0F1-type ATP synthase subunit d, mitochondrial	HPODL_04372	2246	1888	0,84	0,19
F0F1-type ATP synthase subunit h, mitochondrial	HPODL_02780	1020	939	0,92	0,54

Table S13. Expression of homologs of telomerase deletion signature genes of *Saccharomyces cerevisiae* described in Nautiyal et al (2002).

**Function**	**Gene ID** (*S. cerevisiae*)	**Gene ID** (*H. polymorpha*)	**Normalized coverage**			
			**WT**	**D_TER**	**Fold change**	***P* value**
Ribosomal RNA small subunit methyl transferase EMG1	YLR186W	HPODL_00411	104	82	0,79	0,18
Ribosomal protein RPL37A	YLR185W	missing				
Cyclin-dependent kinase activating kinase CAK1	YFL029C	HPODL_03608	193	160	0,83	0,22
Putative cruciform DNA-binding protein CRP1	YHR146W	missing				
Hypothetical protein	YHR115C	HPODL_04281	269	309	1,15	0,35
Subunit of rapamycin-sensitive complex TORC1 involved in growth control	YHR186C	HPODL_00261	145	277	1,90	5 e-05
Hypothetical protein	YJL118W	missing				
Exosome complex component RRP43	YCR035C	HPODL_01168	270	195	0,72	0,03
Protein BUD31	YCR063W	missing				
Glycosidase CRH1	YLR213C	HPODL_03746	297	644	**2,17**	2 e-07
Hypothetical protein	YHT018C	missing				
Integral membrane protein	YHR181W	HPODL_04038	273	197	0,72	0,03

Nautiyal S, DeRisi JL, Blackburn EH: **The genome-wide expression response to telomerase deletion in *Saccharomyces cerevisiae***. *Proc Natl Acad Sci U S A* 2002, **99(14):** 9316-9321.
